# Unintentional Levothyroxine Ingestion by a Child: A Case Report

**DOI:** 10.7759/cureus.78056

**Published:** 2025-01-27

**Authors:** Dinesh V Hinge, Mukund Raizada, Shantanu Gomase, Swati Khapekar, Chaitanya Kumar Javvaji

**Affiliations:** 1 Pediatrics, Jawaharlal Nehru Medical College, Datta Meghe Institute of Higher Education and Research, Wardha, IND; 2 General Medicine, Jawaharlal Nehru Medical College, Datta Meghe Institute of Higher Education and Research, Wardha, IND; 3 Pediatric Cardiology, Jawaharlal Nehru Medical College, Datta Meghe Institute of Higher Education and Research, Wardha, IND

**Keywords:** children, levothyroxine, prednisolone, propranolol, tachycardia

## Abstract

Hypothyroidism is a common endocrine condition in the adult and pediatric population. Levothyroxine, used for the treatment of hypothyroidism, is one of the easily available drugs in any household as either parents or sometimes children are on levothyroxine treatment. Accidental levothyroxine ingestion is increasing nowadays. Unintentional thyroxine ingestion in children may follow diverse manifestations, ranging from asymptomatic presentation to thyrotoxic crisis. While asymptomatic children can be monitored at home, children with severe symptoms need intensive care support. A child can develop convulsions, temperature instability, and arrhythmias, among others. Here, we present the case of a three-year-old male child with a history of accidental ingestion of 3.2 mg levothyroxine. He was initially asymptomatic and developed symptoms such as tachycardia and high blood pressure after 24 hours of ingestion. He was treated with oral propranolol and prednisolone and discharged after five days. After one month, thyroid function was documented as normal.

## Introduction

Accidental drug ingestion is a common cause of morbidity and mortality in the pediatric age group. Common medication intoxications are reported to be due to paracetamol, antibiotics, and antidepressants, among others. Levothyroxine poisoning in children is rare but worrisome for treating doctors and caretakers. The toxic dose of levothyroxine in pediatric age groups is 3 mg/day [1]. Clinical manifestation is variable and is independent of the ingested levothyroxine dose [2]. As few pediatric cases of levothyroxine intoxication are published in the literature, limited information is available on its clinical manifestation and management [3]. Accidental ingestion of high-dose levothyroxine may remain asymptomatic or may present with symptoms such as fever, insomnia, irritability, tremors, tachycardia, hypertension, and convulsions [4]. Sometimes, it can lead to life-threatening complications such as arrhythmias, seizures, thyrotoxic storms, and coma [5]. Treatment of levothyroxine intoxication mainly depends on clinical manifestations in patients and needs close clinical monitoring supported by serial laboratory investigations to look for complications. Treatment options include gastric decontamination, propranolol, steroids, benzodiazepines, and antiepileptic drugs such as phenobarbital, depending on the clinical presentation. One may consider the use of propylthiouracil and occasionally plasmapheresis to remove excess levothyroxine in cases of severe life-threatening symptoms [6]. Here, we present the case of a three-year-old boy with unintentional levothyroxine ingestion (3.2 mg). He was documented to have tachycardia and high blood pressure and was managed successfully with gastric decontamination, steroids, and propranolol.

## Case presentation

**Figure 1:**
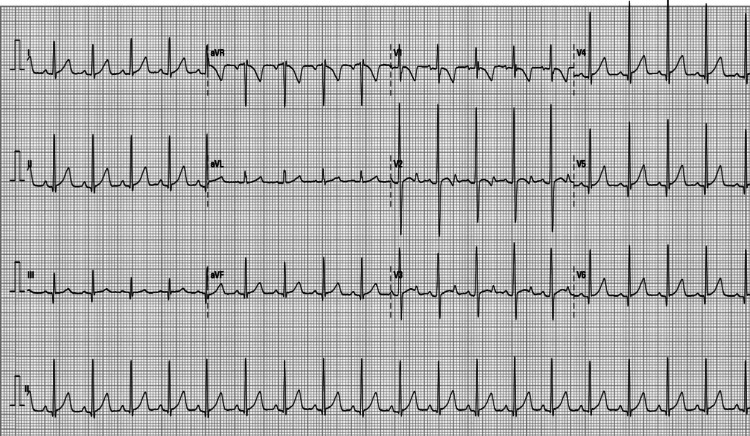
Electrocardiogram on admission to the hospital.

A three-year-old male child was admitted with a presumed history of accidental ingestion of levothyroxine tablets from his father’s medication box. His father was on levothyroxine 100 µg tablet once daily for hypothyroidism. An ingested dose of levothyroxine (3.2 mg) was calculated from the missing tablets in the container. He had ingested around 32 tablets of Thyronorm (100 µg) four hours before presentation to the hospital and was asymptomatic on presentation. The parents denied any vomiting, fever, pain in the abdomen, convulsions, or palpitations. The child was born full-term with a birth weight of 2.8 kg and had an uneventful perinatal period. His development was appropriate for his age in all domains. There was no significant past medical and surgical history. On presentation, he was afebrile (98.3°F), his heart rate (HR) was 110 beats/minute, with a normal respiratory rate (RR) (22/minute), normal blood pressure (BP) (94/68 mmHg), and saturating 98% on room air. His systemic examination, including cardiovascular and neurological examination, was within normal limits. After admission to the hospital, gastric lavage with normal saline was performed. His baseline investigations performed on hospitalization included a complete blood count, liver function test, renal function test, thyroid function test (TFT), lactate dehydrogenase (LDH), and creatine kinase-myoglobin binding (CKMB) which were within normal limits. An electrocardiogram (ECG) done on admission was documented as normal (Figure 1).

**Figure 2:**
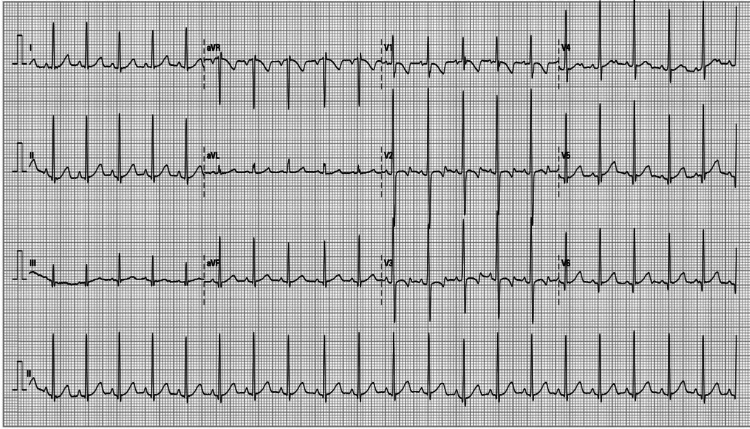
Electrocardiogram changes after 24 hours of levothyroxine ingestion.

An ECG repeated after 24 hours of admission to look for arrhythmias revealed sinus tachycardia and age-appropriate changes (Figure 2).

His TFT was done on admission and monitored serially, as shown in Table 1.

**Table 1:** Serial thyroid function profile after levothyroxine ingestion. T3: triiodothyronine; T4: thyroxine; TSH: thyroid-stimulating hormone

Thyroid parameter	Normal range for 3 years	After 4 hours (on admission)	After 24 hours	After 48 hours	After 72 hours	After 7 days	After 30 days
Total T3(ng/dL)	105–269	171	310	250	178	180	170
Total T4 (µg/dL)	5.9–13.9	>30	>30	>30	25.1	12.8	10
TSH (mIU/L)	0.70–5.97	1.483	0.093	0.039	0.034	0.1	1.2

On admission, his T4 level was high, which was consistent with the history of levothyroxine ingestion. After 24 hours of admission, he developed tachycardia and higher blood pressure (>95th centile for age), and his TFT revealed raised T3, T4, and decreased thyroid-stimulating hormone. We commenced oral propranolol (2 mg/kg/day) and prednisolone (2 mg/kg/day). His HR and BP stabilized after three days. His HR, temperature, RR, BP, blood sugar, and weight were monitored during his hospital stay. All vital parameters, ECG, and blood sugar remained within the normal range for his age after commencing propranolol and prednisolone. The child was discharged on the fifth day of ingestion as T4 and T3 levels on day 4 showed a decreasing trend, and his HR and BP remained normal. He was discharged with safety advice to his parents and with a plan for clinical review and repeated TFT after two days (day 7 of ingestion) and 30 days.

## Discussion

Thyrotoxicosis is an excess of circulating thyroid hormone that causes symptomatic overactivity with an increased metabolic rate [5]. The common causes of thyrotoxicosis include Graves’ disease, thyroiditis, multinodular goiter, and exogenous levothyroxine hormones. In our case, the cause of thyrotoxicosis was accidental ingestion of levothyroxine tablets. Overdose due to accidental ingestion is quite common in children. In adults, the overdose is extremely rare and can be intentional due to mental health issues [7]. Levothyroxine overdose may manifest with symptoms such as fever, increased sweating, flushing, tremors, loose stools, vomiting, convulsions, hypertension, and irregular heart rate [8]. Golightly et al. published a study of 41 children aged one to five years. Overdose of levothyroxine in children generally causes mild or no symptoms. Mild symptoms such as gastric upset, fever, tachycardia, and hyperactive behavior resolve within two weeks without treatment [3]. In a case report, a 2.5-year-old child developed fever, agitation, irritability, increased thirst, tachycardia, and hypertension after ingestion of a massive dose of levothyroxine (10.5 mg). He also developed delayed symptoms such as hair loss and desquamation of the palms and soles after 10 days which were managed symptomatically [7]. In another case report, a 21-year-old female developed transient loss of consciousness and atrial fibrillation after ingesting 10 mg of levothyroxine and was treated with propylthiouracil, steroids, and hemoperfusion to increase levothyroxine clearance. She recovered well and was discharged without any long-term sequel [6]. The onset or severity of symptoms is independent of the dosage of levothyroxine consumed and does not correlate with plasma thyroxin levels. Symptoms may last up to 6 to 11 days [7].

## Conclusions

Children have easy access to levothyroxine tablets at home as they, or adults, may take them regularly as medication. It has a good color, smell, and taste which makes it attractive to children. If a child takes levothyroxine as prescribed medication, it should be given by a caretaker or directly supervised if the child is relatively older and self-administering it. Children should be taken to the hospital urgently if there is a suspicion of accidental ingestion. Children need hospitalization for clinical and laboratory monitoring for signs of toxicity and hyperthyroidism. Parents should be vigilant and should keep all medications out of reach of children to avoid accidental ingestion and intoxication, as consequences can be grave, with significant morbidity and mortality.
